# Anthocyanins from *Malus* spp. inhibit the activity of *Gymnosporangium yamadae* by downregulating the expression of *WSC*, *RLM1*, and *PMA1*

**DOI:** 10.3389/fmicb.2023.1152050

**Published:** 2023-05-03

**Authors:** Yu Wang, Hong An, Yan-Nan Guo, Qian Wang, Yuan-Yuan Shang, Ming-Kun Chen, Yi-Xin Liu, Jia-Xin Meng, Shuang-Yu Zhang, Jun Wei, Hou-Hua Li

**Affiliations:** College of Landscape Architecture and Art, Northwest A&F University, Yangling, Shaanxi, China

**Keywords:** anthocyanins, *Gymnosporangium yamadae*, teliospores, transcriptome, cell wall, cell membrane

## Abstract

*Malus* plants are frequently devastated by the apple rust caused by *Gymnosporangium yamadae* Miyabe. When rust occurs, most *Malus* spp. and cultivars produce yellow spots, which are more severe, whereas a few cultivars accumulate anthocyanins around rust spots, forming red spots that inhibit the expansion of the affected area and might confer rust resistance. Inoculation experiments showed that *Malus* spp. with red spots had a significantly lower rust severity. Compared with *M. micromalus*, *M.* ‘Profusion’, with red spots, accumulated more anthocyanins. Anthocyanins exhibited concentration-dependent antifungal activity against *G. yamadae* by inhibiting teliospores germination. Morphological observations and the leakage of teliospores intracellular contents evidenced that anthocyanins destroyed cell integrity. Transcriptome data of anthocyanins-treated teliospores showed that differentially expressed genes were enriched in cell wall and membrane metabolism-related pathways. Obvious cell atrophy in periodical cells and aeciospores was observed at the rust spots of *M.* ‘Profusion’. Moreover, *WSC*, *RLM1*, and *PMA1* in the cell wall and membrane metabolic pathways were progressively downregulated with increasing anthocyanins content, both in the *in vitro* treatment and in *Malus* spp. Our results suggest that anthocyanins play an anti-rust role by downregulating the expression of *WSC*, *RLM1*, and *PMA1* to destroy the cell integrity of *G. yamadae*.

## Introduction

Apple rust, caused by *Gymnosporangium yamadae* (Yun et al., 2009), is a severe foliage disease that directly affects the growth and production of apple trees (Lee et al., 2016; Lu et al., 2017; Duan et al., 2019). This pathogen is a Basidiomycotina fungus with parasitic characteristics, and is difficult to cultivate artificially. It forms pycnospores and aeciospores on *Malus* spp. and is then transferred to *Sabina chinensis*, where the teliospores are formed (Yun et al., 2009; Duan et al., 2019). Teliospores germinate and release basidiospores to infect apple tree leaves. *G. yamadae* lacks a urediniospore stage in the life history cycle, with only four spore types, pycnospores, aeciospores, teliospore, and basidiospores. Thus *G. yamadae* does not have the ability to repeatedly infect its hosts within a single life history cycle, unlike other rust fungi with urediniospores (Tao et al., 2019). There are only limited studies on the biological control of rust pathogens. Xue et al. (2005) reported that *Peganum multisectum* extracts could effectively inhibit the activity of wheat stripe rust with 78% inhibition. Ye et al. (2013) reported that the fruit extracts of *Daphne giraldii nitsche* and *Siberian Coeklebu* had inhibitory effects on rust sporangium germination and mycelia growth of wheat stripe rust. It was also found that *Trichoderma harzianum* could be used as a biocontrol agent against the leaf rust pathogen of the medicinal plant *Justicia gendarussa* (Ragi et al., 2013). When infected with rust, colored susceptible tissues are produced in apple tree leaves, which become defensive against rust by detoxification (Mierziak et al., 2014; Peixoto et al., 2016). Field observations revealed that most *Malus* species and cultivars produce yellow spots while few cultivars produce red spots; the disease is more serious in the former and mild in the latter. Artificial inoculation tests produced results consistent with those of field observations, and the incidence, rust index, and lesion area ratio were lower in *Malus* spp. with red rust spots than in those with yellow rust spots. Our previous research indicated that these red spots were due to the formation and accumulation of anthocyanins (Liu et al., 2019). However, it is unclear whether anthocyanins play an essential role in anti-rust infections.

Anthocyanins belong to the flavonoid family and are the main coloring substances in the petals, leaves, and fruits of plants (Veitch and Grayer, 2011; Yang et al., 2019; Han et al., 2020; Fu et al., 2021; LaFountain and Yuan, 2021; Ma et al., 2021). In addition to the colouration effect, anthocyanins also have potent inhibitory effects on various fungi, such as *Botrytis cinerea* (Jiang et al., 2017), *Geotrichum candidum*, and *Candida albicans* (Wen et al., 2016). Anthocyanins are natural antimicrobials that are safe and effective. Antimicrobial mechanisms have been reported to include damage to cell wall integrity (Liu et al., 2015; Yang L. et al., 2016 Sun et al., 2017; Li et al., 2020; Deng et al., 2021), changes in cell membrane permeability (Chen et al., 2018; Zhao et al., 2019; Bautista-Silva et al., 2020; Wang et al., 2020; Wu et al., 2020), induced reactive oxygen species stress (Yang et al., 2021), cell apoptosis (Lv et al., 2020), and inhibition of the cell growth cycle (Wu et al., 2012). However, it is still unknown whether anthocyanins in *Malus* spp. inhibit the activity of *G. yamadae*, and there are no reports on the possible antifungal mechanisms of anthocyanins against this species.

In the present study, we investigated the inhibitory effects of anthocyanins on *G. yamadae*. The mode of anthocyanin inhibition was explored via microscopic observations, physiological assays, transcriptome sequencing, and gene expression analysis in *Malus* spp.

## Materials and methods

### Plant materials and treatment

Ten-year-old adult trees of six *Malus* species (Table 1) were used as the experimental materials. Trees were grown in the crab apple germplasm nursery at Northwest A&F University, Yangling, China. The trees were infected with *G. yamadae* by artificial inoculation. Unless otherwise specified, the inoculated fungal solution was a teliospore suspension (1 × 10^6^ mL^−1^, 100 μL) treated with double distilled water (ddH_2_O) for 12 h. The germplasm nursery was not exposed to any pesticides during the experimental period. Uninfected leaf tissues (UITs) and rust-infected leaf tissues (RITs) were collected at different developmental stages after rust infection.

**Table 1:** Rust infection severity in six *Malus* spp. accessions.

Accession	Rust spot color	Incidence (%)	RI	Lesion area ratio (%)
*M. micromalus*	Yellow	94.33 ± 0.58a	45.40 ± 4.50a	34.73 ± 14.41a
*M. melliana*	Yellow	92.78 ± 0.41b	42.36 ± 3.77a	31.69 ± 9.46b
*M.* ‘Snow Drift’	Yellow	89.85 ± 0.72a	36.85 ± 3.62b	27.78 ± 5.86c
*M.* ‘Profusion’	Red	72.17 ± 0.57c	16.11 ± 0.78 cd	6.79 ± 1.31e
*M.* ‘Radiant’	Red	84.88 ± 1.02b	21.53 ± 0.55c	8.84 ± 0.97e
*M.* ‘Strawberry Parfait’	Red	78.95 ± 1.35c	19.64 ± 1.65c	12.75 ± 3.32d

### Assessment of rust severity

Rust severity was assessed using the method described by Zhao et al. (2011) with slight modifications. Five trees of each *Malus* spp. were sampled as one replicate for further tests, at least three replicates were performed, and a total of 100 leaves artificially inoculated with rust from each tree were collected. Incidence, rust index, and lesion area ratio were calculated. Incidence was calculated as a percentage of the total number of leaves with rust spots, i.e., RITs. The rust index (RI) was then scored based on the number of RITs on a 0–9 scale: 0, no RITs; 1, 1–10 RITs; 3, 11–30 RITs; 5, 31–50 RITs; 7, 51–70 RITs; and 9, number of RITs >71. The RI was calculated using Eq. (1):


(1) $$\mathrm{RI}=\frac{\sum \left(\mathrm{rust score}\times \mathrm{leaves number}\;\mathrm{at}\;\mathrm{this grade}\right)}{\mathrm{total number of leaves}\times \mathrm{the largest score}}\times 100\%\;\;$$

The lesion area ratio was calculated by using Adobe Photoshop CS6 software.

### Determination of total anthocyanins content

First, 0.1 g lyophilized leaf sample was weighed and soaked in 10 mL extraction solution (97:3 methanol: hydrochloric acid). The samples were then incubated at 4°C for 48 h in the dark and extracted using ultrasound for 1 h. Next, we centrifuged the mixture at 6,000 rpm for 3 min and collected the supernatant. Total anthocyanins content was determined by measuring absorbance at 530 and 657 nm (A_530_ and A_657_, respectively) on a UV-3802 spectrophotometer (Unico, Dayton, OH, United States) and applying the following formula: A_530_–0.25 × A_657_. Cyanidin chloride [> 95% high-performance liquid chromatography (HPLC) grade; Sigma-Aldrich, St. Louis, MO, USA] was used to generate calibration curves. Finally, total anthocyanins content was normalized to the dry weight of each sample.

### HPLC-diode array detector analysis of anthocyanin components

The collected eluate sample was passed through a 0.45 μm filter and its composition was detected using HPLC-DAD analysis on a Shimadzu LC-2030C Liquid Chromatograph (Shimadzu Corp., Kyoto, Japan). The results of each component were compared with the retention time and standard curve of each reference substance. The respective structures were confirmed by comparison with a standard using liquid chromatography/mass spectroscopy (LC/MS), as described by Li et al. (2007). Three biological replicates were analyzed.

### *In vitro* germination of *Gymnosporangium yamadae* teliospores

Ungerminated teliospores were collected from *S. chinensis* in the crab apple germplasm nursery of Northwest A & F University. Purified anthocyanins from *Malus* spp. were used (Liu et al., 2022). Teliospores of *G. yamadae* (1 × 10^6^ mL^−1^, 100 μL) were inoculated into 20 mL solutions containing 0.00 (control), 0.125, 0.25, 0.375, 0.50, or 1.00 mg mL^−1^ anthocyanins. When the length of the basidium was greater than half the diameter of the teliospore, the teliospore was considered germinated. The germination percentage was estimated by counting the number of germinated teliospores after 12 h in five microscope fields.

### Fluorescence microscopy observation

Cell viability was examined using 50 mg L^−1^ fluorescein diacetate (FDA; Cat No. F7378; Sigma-Aldrich) and 10 mg L^−1^ propidium iodide (PI; Cat No. P3566; Thermo Fisher Scientific, Waltham, MA, United States); After staining, the *G. yamadae* teliospores were examined using fluorescence microscopy. Three independent experiments were performed.

### Determination of alkaline phosphatase activity

The alkaline phosphatase (AKP) activity of *G. yamadae* teliospores was determined using the AKP kit (Solarbio Science and Technology Co., Ltd., Beijing, China). One unit of AKP activity was defined as the time taken (min) to produce 1 μmol phenol per 1 g *G. yamadae* teliospore sample at 37°C. Each experiment was repeated thrice.

### Determination of cellular content leakage

*G. yamadae* teliospores treated with anthocyanins and ddH_2_O for 0–12 h were used to determine the leakage of cellular contents. The supernatant was harvested by centrifugation at 1,100 ×g for 5 min. Leakage of nucleic acids and proteins from *G. yamadae* cells was evaluated using an ultra-violet (UV) spectrophotometer (Shimadzu Corp.). The electrical conductivity was measured using a DDS-307 conductivity meter (Shimadzu Corp.). Each experiment was repeated thrice.

### RNA extraction, cDNA sequencing, and analysis of differentially expressed genes

*G. yamadae* teliospore samples treated for 12 h with 0.5 mg mL^−1^ anthocyanin solution or ddH_2_O (control) were used for this experiment. Total RNAs were extracted using the TRIzol reagent (Invitrogen, Carlsbad, CA, United States). RNA purity was checked using a NanoPhotometer® spectrophotometer (IMPLEN, CA, United States) and RNA concentration was measured using Qubit® RNA Assay Kit and a Qubit® 2.0 Fluorometer (Life Technologies, CA, United States). RNA integrity was assessed using the RNA Nano 6,000 Assay Kit of the Bioanalyzer 2,100 system (Agilent Technologies, Inc., Santa Clara, CA, United States). RNA degradation and contamination were monitored by electrophoresis on 1% agarose gels. cDNA library construction and RNA-sequencing (RNA-seq) were performed using the Huada DNBSEQ platform (Shenzhen, China). Genes with log2 (Fold Change) > 0.5 and an adjusted *p*-value (padj) < 0.05, as determined by DESeq2, were assigned as DEGs (Love et al., 2018; Yin et al., 2021).

### Gene ontology and Kyoto Encyclopedia of Genes and Genomes enrichment analyses of DEGs

Gene ontology (Go) enrichment analysis of DEGs was performed using the Cluster Profiler R package (Mao et al., 2005). GO terms with padj <0.05 were considered significantly enriched (Young et al., 2010). To identify the biological pathways active in *G. yamadae*, all DEGs were mapped to the reference canonical pathways contained in the KEGG pathway database. Statistical enrichment of DEGs in KEGG pathways was also tested using the Cluster Profiler R package (Mao et al., 2005).

### Quantitative real-time-PCR validation

To validate the reliability of the data obtained from the transcriptome sequencing (i.e., RNA-seq) of *G. yamadae*, a qRT-PCR was conducted on 18 DEGs using *ARF1*(*CL5610.Contig2_All*) as the internal reference (Supplementary Table 1). For the *in vivo* validation of *Malus* spp., 18S was used as the internal reference gene (Supplementary Table 1), and RNA samples from RITs at different stages were used. The relative expression of each gene toward the reference gene was calculated using the 2^-ΔΔCt^ method (Liu et al., 2015) with three independent replicates.

### Statistical analysis

All data were analyzed using SPSS software (version 20.0; IBM SPSS Inc., Chicago, IL, United States) and are presented as mean values ± standard deviation. All experimental data analyses were performed using one-way analysis of variance (ANOVA), and *p* < 0.05 was defined as statistically significant. All graphs were plotted using GraphPad Prism (version 8; GraphPad Software, San Diego, CA, United States).

## Results

### Evaluation of rust infection severity in *Malus* spp.

Six *Malus* spp. accessions were evaluated for their rust response (Table 1). All accessions were artificially inoculated with *G. yamadae*, and spot-like rust began to appear on the foliage 11 days after inoculation. The spots gradually became larger and displayed a red or yellow color. The rust infection severity of *Malus* spp. was determined 22 days after inoculation. No accession was immune to the disease, with an incidence ranging from a maximum of 94.37 ± 2.12% to a minimum of 71.80 ± 8.41%. RI also varied significantly among the six *Malus* spp. accessions. The mean RI of *Malus* spp. with red rust spots was 18.46, whereas that of *Malus* spp. with yellow rust spots was 40.62, i.e., almost twice as high as that of *Malus* spp. with red rust spots. We also observed that the size of individual lesion areas in *Malus* spp. with red rust spots was significantly smaller than that in *Malus* spp. with yellow rust spots at the same inoculation time, and that lesions developed more slowly. The average lesion area ratio for *Malus* spp. with red rust spots was 9.24%, which is a small percentage of the leaf area. In contrast, the lesion area ratio for *Malus* spp. with yellow rust spots was 31.05%, almost 1/3 of the leaf area. *Malus* spp. with rust spots of different colors showed different rust resistances. Among the six accessions, *M.* ‘Profusion’ with red rust spots was the most resistant to rust, whereas *M*. *micromalus* with yellow rust spots was the most sensitive to rust.

### Analysis of total anthocyanins content and composition

**Figure 1:**
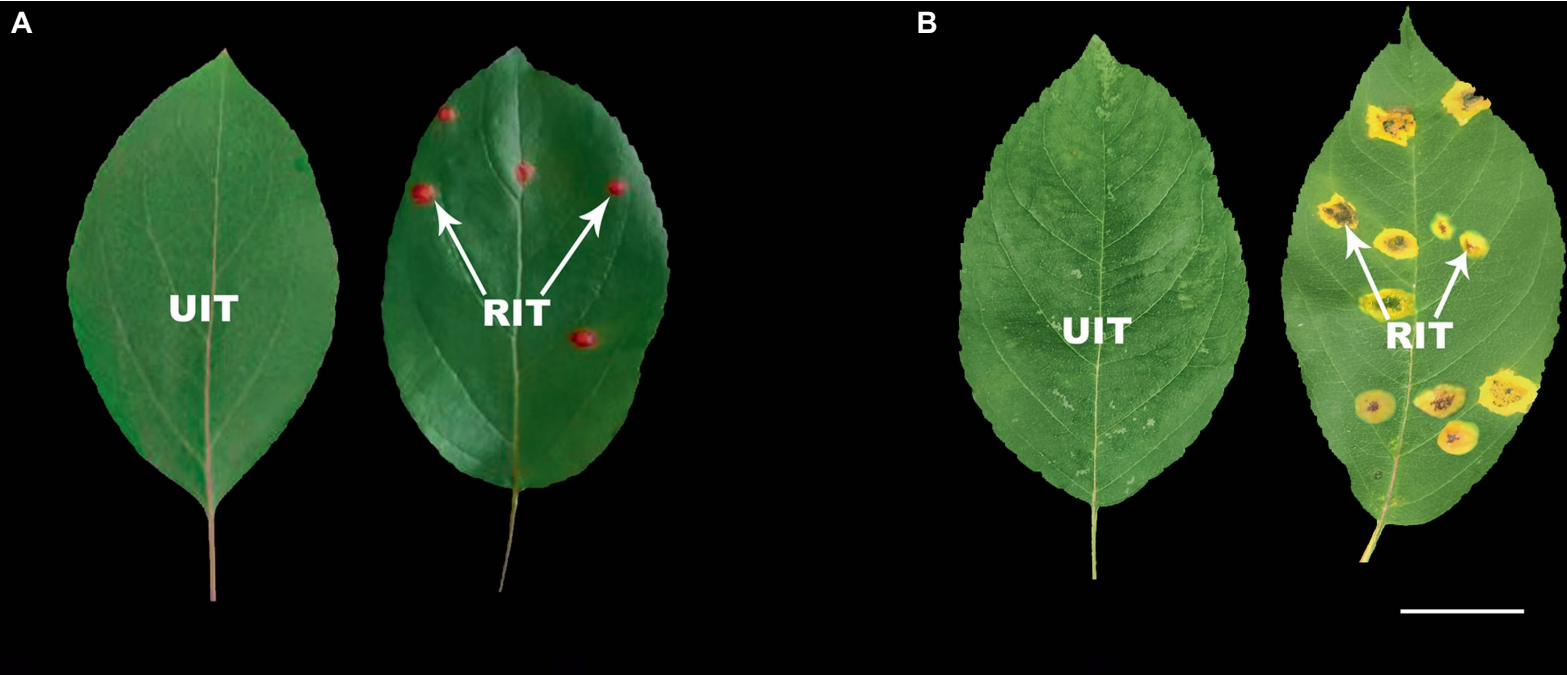
Rust infection severity in two *Malus* spp. **(A)** *Malus* ‘Profusion’. **(B)** *Malus micromalus.* RIT, rust-infected leaf tissue; UIT, uninfected leaf tissue. Scale bars: 2 cm.

The *Malus* spp. accessions that were determined 22 days after inoculation as most susceptible (*M.* ‘Profusion’) and most resistant (*M*. *micromalus*) to rust were selected for anthocyanin content analysis (Figure 1). The UITs of the two accessions served as controls for the anthocyanin analysis. The total anthocyanins content of RITs in *M.* ‘Profusion’ was significantly increased and was 11.39 times greater than that in UITs (Table 2). In contrast, the total anthocyanins content in *M*. *micromalus* was minimal, and there was no significant difference between RITs and UITs.

**Table 2:** Type and content of anthocyanin biosynthesis-related compounds in RITs and UITs of two *Malus* spp.

Compound	*M.* ‘Profusion’		*M. micromalus*	
	UIT/mg.kg^−1^	RIT/mg.kg^−1^	UIT/mg.kg^−1^	RIT/mg.kg^−1^
Total anthocyanins content	116.67 ± 0.11b	1328.67 ± 32.23a	8.82 ± 0.77a	9.23 ± 0.62a
Cyanidin-3-galactoside chloride	97.52 ± 7.35b	1198.58 ± 21.64a	8.10 ± 0.44a	8.29 ± 0.31a
Cyanidin-3-O-rutinoside	6.57 ± 0.43b	17.01 ± 1.47a	0.37 ± 0.02a	0.41 ± 0.05a
Cyanirin-3-O-arabinoside	2.96 ± 0.15b	5.12 ± 0.52a	ND	ND
Cyanidin-3,5-O-diglucoside	ND	1.23 ± 0.08a	ND	ND

Data are presented as the mean ± standard deviation (SD) of three biological replicates. The letters represent significant differences between the UITs and RITs, as judged by the paired *t*-test (*p* < 0.05).

The HPLC-DAD results were compared to the UV absorption curves and retention times of the standards. The substances with the maximum A_520_ belonged to anthocyanosides. Cyanidin-3-galactoside chloride, cyanidin-3-O-rutinoside, cyanirin-3-O-arabinoside, and cyanidin-3,5-O-diglucoside were detected in *M.* ‘Profusion’ (Table 2). These centaureidin glycosides presented a total content similar to that of total anthocyanins, with cyanidin-3-galactoside chloride accounting for 83.58 and 90.21% of the total anthocyanins content in UITs and RITs, respectively. Levels of cyanidin-3-O-rutinoside, cyanirin-3-O-arabinoside, and cyanidin-3,5-O-diglucoside were extremely low. Of these, cyanidin-3,5-O-diglucoside was detected only in RITs. In *M*. *micromalus*, only two anthocyanins, cyanidin-3-galactoside chloride and cyanidin-3-O-rutinoside, were detected (Table 2), with no significant differences between RITs and UITs. This considerable difference in anthocyanins content may account for the different rust susceptibility patterns between the two accessions.

### Anthocyanins inhibited the germination of *Gymnosporangium yamadae* teliospores

**Figure 2:**
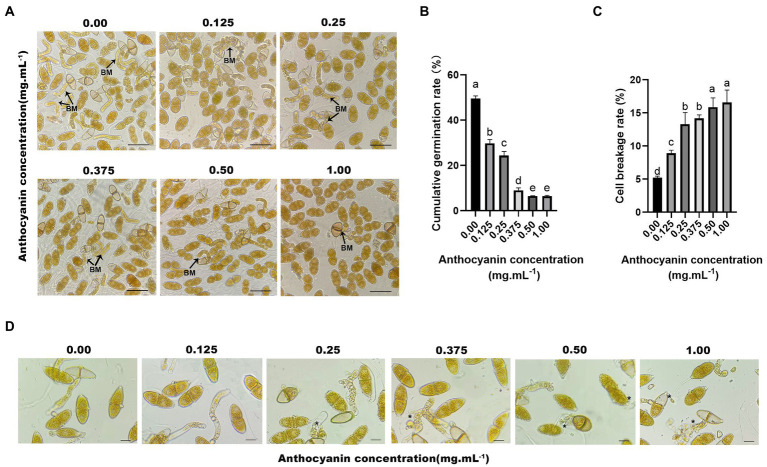
Anthocyanins inhibit the germination of *G. yamadae* teliospores. **(A)** Teliospores treated with different concentrations of anthocyanins, BM: basidium. **(B)** Cumulative germination rate of teliospores. Each value is the mean of three replicates. The vertical bar indicates the standard error. a–e Statistically significant differences between control and treatments. **(C)** Cell breakage rate of teliospores. Each value is the mean of three replicates. The vertical bar indicates the standard error. a–e Statistically significant differences between control and treatments. **(D)** Morphological effects of anthocyanins on teliospores. Asterisks indicate broken spores after anthocyanins treatment. Scale bars: **(A)** 60 μm; (D) 25 μm.

We selected anthocyanins purified from *Malus* spp. for *in vitro* fungal inhibition assays. The purity of the anthocyanin sample was 69.4%, and the major composition is cyanidin-3-galactoside chloride (Liu et al., 2022). Anthocyanins from *Malus* spp. strongly affected the germination of *G. yamadae* teliospores (Figure 2A), and the germination rate of teliospores decreased with increasing anthocyanins concentration. At 0.50 and 1.00 mg mL^−1^, the number of germinated teliospores was low, and germination rates were 6.51 and 6.48%, respectively (Figure 2B). Thus, anthocyanins had a dose-dependent inhibitory effect on teliospore germination. In addition, we also found that with the increase of anthocyanin concentration, the cell breakage rate of fungal spores gradually increased, reaching a maximum of 15.83% (Figure 2C). When teliospores suspension treated with anthocyanins were inoculated on *Malus* spp., both *M*. *micromalus* and *M.* ‘Profusion’ became significantly less susceptible to rust. The highest incidence were 17.37 and 10.60% for *M*. *micromalus* and *M.* ‘Profusion’, respectively (Supplementary Figure 1). Subsequent observations revealed that although teliospores suspension treated with anthocyanins continued to infect *Malus* spp., most of the rust spots grew to a certain extent and became dry, and only a few developed normal pycnium and aecidium.

### Morphometric analysis of teliospores

Microscopic observations revealed that the appearance and structure of *G. yamadae* changed with anthocyanins treatment (Figure 2D). In control samples, the intracellular organization of cells exhibited unambiguous and regular morphology and plump and uniform distribution of cytoplasm. In contrast, the anthocyanin-treated teliospore cells were damaged to varying degrees. Moreover, the higher the concentration of anthocyanins, the more severe the cell destruction. The teliospores treated with 0.125 mg mL^−1^ anthocyanin showed no significant change compared to the control. In the treatment using 0.25 mg mL^−1^ anthocyanin, the cell morphology of teliospores was destroyed, some cells shrank, and cellular contents leaked. In the treatments using 0.50 and 1.00 mg mL^−1^ anthocyanins, the cell damage was even more severe; the cells were extensively shrunk, leakage of cellular contents increased, and the basidiums were deformed and distorted, which resulted in irregular shrinkage or even interruption. Thus, microscopic evidence suggests that anthocyanins disrupt the cellular structure of teliospores, disrupting cell integrity, causing leakage of cellular contents and deformation of the basidium.

### Anthocyanins treatment destroyed teliospore cell integrity

**Figure 3:**
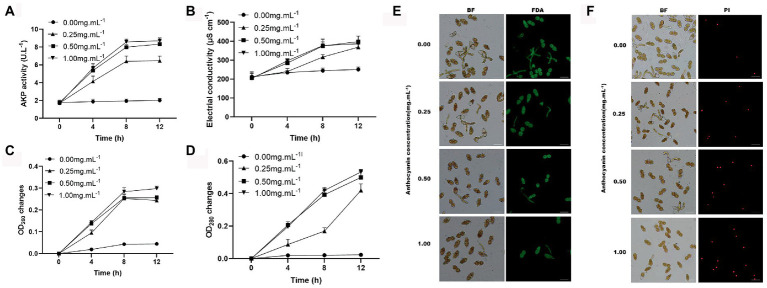
The effects of anthocyanins on *G. yamadae* cell integrity. **(A)** The extracellular AKP activities. **(B)** Electrical conductivity. **(C)** nucleic acids content. **(D)** protein concentration. **(E)** FDA staining assay. **(F)** PI staining assay. Data are mean ± standard error of three replicate samples. Vertical bars represent standard errors of the means. Scale bars: **(E,F)** 60 μm.

The use of FDA to assess cell viability in the teliospores revealed that control teliospores were easily stained by this chemical in high fluorescence intensity, whereas the teliospores treated with anthocyanins were rarely stained. In fact, only individual teliospores treated with 0.50 and 1.00 mg mL^−1^ anthocyanins were stained (Figure 3E). The permeability of the cell wall and membrane of teliospores was examined. AKP is an essential enzyme in the cell wall and membrane. When the cell wall is destroyed, AKP leaks out; hence, AKP activity can be detected extracellularly (Yang S. et al., 2016). In the present study, anthocyanins increased the AKP activity of teliospores, and this change became more pronounced over time (*p* < 0.05). At 12 h, the extracellular AKP activities under 0.50 and 1.00 mg mL^−1^ anthocyanins were 8.339 ± 0.36 and 8.704 ± 0.32 U L^−1^, respectively, which was significantly higher than that in the control group (6.47 ± 0.52 U L^−1^) (Figure 3A). This indicated that the permeability of the fungal cell wall was changed or damaged, which increased the AKP content in the fungal solution. PI was used to check membrane integrity in *G. yamadae* teliospore cells. Compared with the control, more cells were stained with PI upon exposure to 0.50 and 1.00 mg mL^−1^ anthocyanins (Figure 3F), indicating that a considerable percentage of cells lost membrane integrity. As PI is membrane-impermeable and generally excluded from viable cells, this result also suggests that the cells presenting red fluorescence (PI) were dead.

Disruption of cellular integrity can lead to the leakage of cellular contents. Fungal cultures were tested for electrical conductivity, protein concentration, and nucleic acid content. A constant increase in the electrical conductivity was observed in the anthocyanin-treated samples over time, which was significantly different from that of the control (Figure 3B). The disruption of cellular integrity was further confirmed by the significant increase in the absorbance of proteins and nucleic acids observed in the supernatant using UV spectrometry (Figures 3C,D). This is consistent with previous microscopic observations of the cellular content leakage. The above results demonstrate that under anthocyanins treatment, the cell integrity of the fungus is destroyed, causing leakage of intracellular components, which significantly affects the normal physiological metabolism of cells.

### Gene expression and enrichment analyses

**Figure 4:**
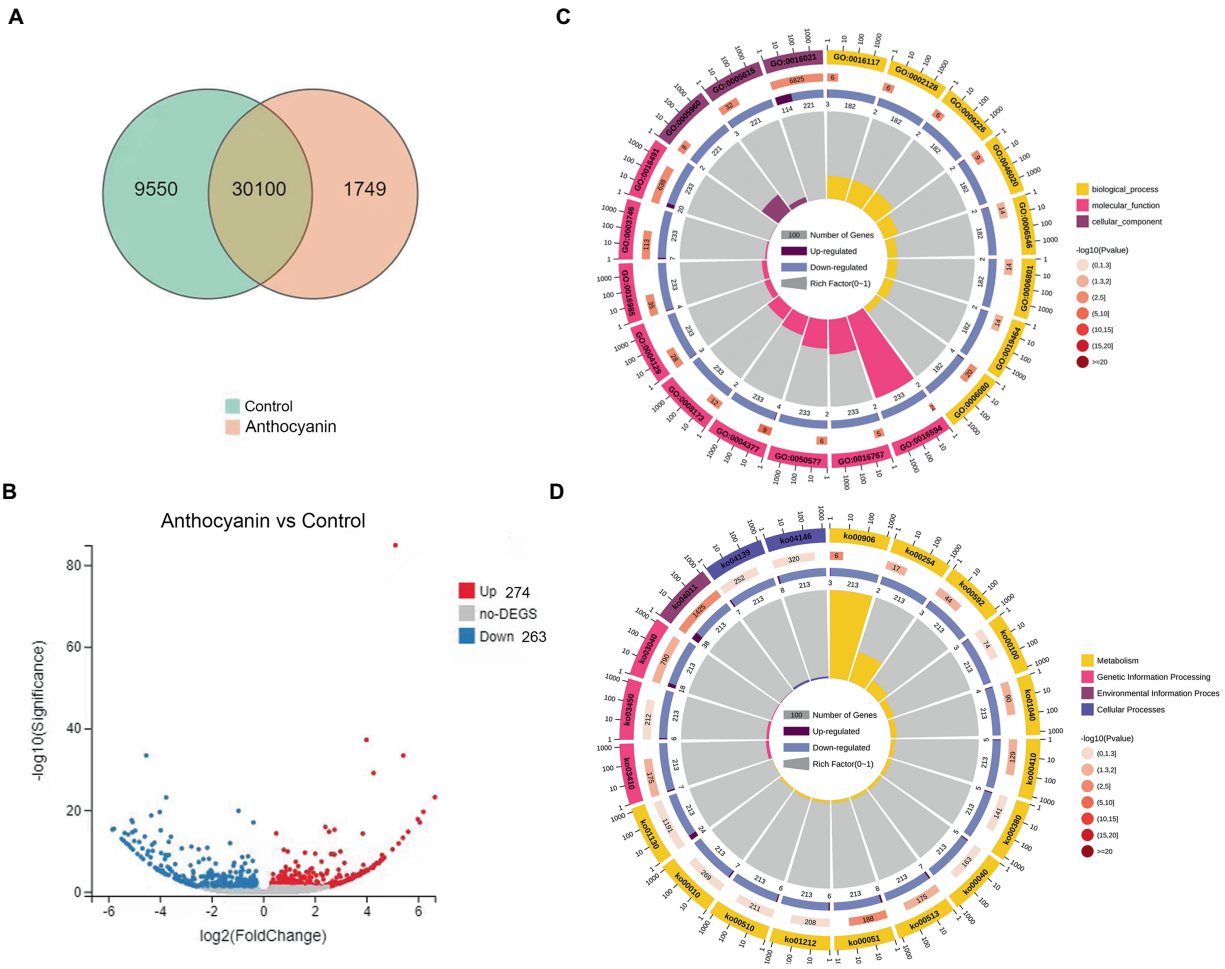
GO and KEGG analyses of DEGs between anthocyanin-treated and control groups. **(A)** Venn diagram showing the overlap of expressed genes. Pink represents anthocyanin treatment, green represents control. **(B)** Volcano plot of DEGs. Splashes represent different genes, where gray indicates the mean number of genes without significant differential expression, red indicates the mean of significantly upregulated genes, and blue indicates the mean of significantly downregulated genes. **(C)** GO enrichment analysis of DEGs. **(D)** KEGG enrichment analysis of DEGs.

To assess the changes in *G. yamadae* gene expression profiles under anthocyanins treatment, the total RNA of *G. yamadae* samples treated with 0.50 mg mL^−1^ anthocyanins and control samples (CK) was extracted for transcriptome sequencing (Supplementary Table 2). There were 31,849 expressed genes in the anthocyanin-treated samples and 39,650 in CK samples. Among them, 30,100 genes were co-expressed across all samples, of which 9,550 and 1749 were specifically expressed in the anthocyanin-treated and CK samples, respectively (Figure 4A). Overall, 537 DEGs were identified between the anthocyanin-treated and CK samples, consisting of 274 upregulated and 263 downregulated genes (Figure 4B). GO and KEGG analyses were conducted for the DEGs found between the two treatments of *G. yamadae*. The top 20 pathways with the most abundant DEGs in the GO and KEGG databases are listed in Supplementary Tables 3, 4, respectively. The results of GO enrichment analysis (Figure 4C) showed that the integral components of the membrane pathways (GO:0016021) were significantly affected by anthocyanins. KEGG enrichment analysis (Figure 4D) showed that the MAPK signaling pathway was significantly enriched (Ko04011), and further analysis revealed that the DEGs were mainly associated with the cell wall integrity pathway (CWI, a type of MAPK cascade pathway). GO and KEGG enrichment analyses showed that anthocyanin stress affected the metabolic activities of the cell wall and cell membrane of *G. yamadae* teliospores. This result is consistent with the microscopic observations.

### Changes of gene expression in the cell wall and membrane metabolic pathways of *Gymnosporangium yamadae* under anthocyanins treatment

The pathways specifically associated with the cell wall were GDP-Man:Man3GlcNAc2-PP-Dol alpha-1,2-mannosyltransferase activity (GO:0004377), substituted mannan metabolic process (GO:0006080), mannan endo-1,4-beta-mannosidase activity (GO:0016985), nucleotide-sugar biosynthetic process (GO:0009226), MAPK signaling pathway (Ko04011), fructose and mannose metabolism (Ko00051), and various types of N-glycan biosynthesis (Ko00513). Among them, we focused on some essential genes related to the MAPK signaling pathway, particularly the CWI pathway. *WSC* (*Unigene2519_All*) and *RLM1* (*CL2152.Contig4_All*) were downregulated after anthocyanins treatment (Supplementary Figure 2). *RLM1* is a crucial transcription factor that encodes most of the transcriptional genes responsible for exporting CWI. *WSC* is a plasma membrane sensor of the CWI pathway and plays an essential role in maintaining the integrity of the cell wall. In addition, most genes in the mannose (the main component of the fungal cell wall) metabolic pathway, such as *MNN2* (*CL4560.Contig3_All*), *MAN* (*CL238.Contig6_All*), *GMPP* (*Unigene2859_All*), *UBP1* (*Unigene4211_All*), *ALG11* (*CL680.Contig6_All*), and *MAN1* (*CL2191.Contig7_All*), were up-regulated after the 0.50 mg mL^−1^ anthocyanins treatment. These results suggest that fungi increase mannose and cell wall stability in response to anthocyanin stress by upregulating gene expression in the mannose metabolic pathway.

Anthocyanins also affect multiple membrane-related metabolic processes in the teliospores, such as the ergosterol biosynthetic process (GO:0006696), integral component of the membrane (GO:0016021), biosynthesis of unsaturated fatty acids (Ko01040), steroid biosynthesis (Ko00100), and fatty acid metabolism (Ko01212). Compared with control samples, membrane-associated DEGs (Supplementary Figure 2), such as mitochondrial phosphate transporter (*CL5029.Contig9_All*, *SLC25A23S* and *CL478.Contig6_All*, *SLC35A1*), proton-transport ATPase (*CL1563.Contig5_All*, *PMA1*), ABC transporters (*CL424.Contig6_All*, *ABCC1*), and nine transmembrane superfamily members (*CL3755.Contig10_All*, *TM9SF2*), and other membrane proteins were downregulated under the anthocyanins treatment. Membrane proteins are responsible for various functions including nutrient transport, response to environmental stress, biofilm formation, and antibiotic resistance (Nakayama et al., 2013; Li et al., 2015). Thus, even slight changes in membrane proteins may negatively affect cell function and result in cell death (Yi et al., 2018). Anthocyanins treatment also upregulated the expression of steroid biosynthesis and ergosterol pathway genes, including *POR* (*CL195.Contig10_All*), *ERG25* (*CL4834.Contig1_All*), *ERG4* (*CL761.Contig5_All*), and *ERG6* (*Unigene109_All*). These genes encode vital enzymes involved in the synthesis of ergosterol, which helps maintain cell membrane stability and fluidity. Similarly, in addition to the downregulation of *SCD* (*CL381.Contig3_All*), other fatty acid synthesis-related genes, such as *ACAA1* (*Unigene1147_All*), *OPR* (*CL1271.Contig15_All*), *FAD2* (*CL115.Contig7_All*), and *fabG* (*CL2696.Contig2_All*), were also upregulated under anthocyanins treatment. Thus, anthocyanins might disrupt the cell membrane of *G. yamadae*, and they can adjust the stability and fluidity of cell membranes by upregulating genes in the ergosterol and fatty acid synthesis pathways, thereby responding to anthocyanins stress.

### Validation of DEGs expression levels

DEGs of the three most enriched metabolic pathways in the GO and KEGG databases were selected to validate the RNA-seq results (Supplementary Figure 3). Ten of them were related to cell wall and membrane metabolism, four were related to the biosynthesis of antibiotics, and four were related to oxidoreductase activity. The qRT-PCR results obtained for the DEGs under the 0.50 mg mL^−1^ anthocyanins and control (0.00 mg mL^−1^) treatments was consistent with transcriptome data, thus confirming the reliability of the RNA-seq data. Expression analysis revealed that five of the DEGs (*WSC*, *RLM1 PMA1*, *ABCC1*, and *SCD*) related to the cell wall and membrane metabolism were all downregulated under different concentrations of anthocyanins, and the downregulation was more pronounced with increasing anthocyanins concentration. The remaining five DEGs (*MAN*, *MAN1*, *ERG4*, *ERG6*, and *FAD2*) showed a trend of first decreasing and then increasing under increasing anthocyanins concentration. The results of the expression analysis were consistent with our previous hypothesis that *G. yamadae* responds to or resists adverse environments by adjusting its gene expression. As the concentration of anthocyanins increases, cells can alleviate the toxic effects of anthocyanins by upregulating the expression of genes related to the cell membrane and wall components. There was no apparent trend in the expression changes of DEGs related to oxidoreductase activity (*NADPH2*, *CL1271.Contig15_All*; *ND2*, *Unigene768_All*; *ARD1*, *CL376.Contig9_All*; and *CAT*, *CL3992.Contig2_All*). The expression levels of the four genes (*RPIA*, *CL907.Contig5_All*; *LRA1*, *Unigene2082_All*; *rocF*, *CL1499.Contig2_All*; and *SOU1*, *CL2696.Contig2_All*) involved in the biosynthesis of antibiotics were higher than those of the control after anthocyanins treatment. Therefore, anthocyanins not only change the cell wall and membrane metabolic pathways of fungi, but may also stimulate fungi to produce antibiotic substances to resist injury effects.

### Observation of leaf tissues of *Malus* ‘Profusion’ and *Malus micromalus* infected with rust

**Figure 5:**
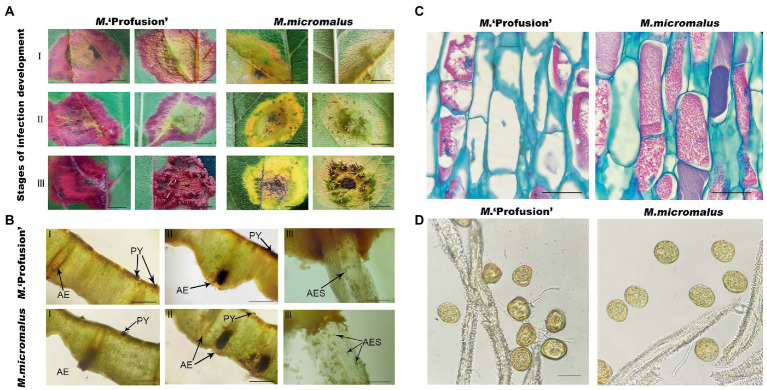
Observation of leaf tissues infected with rust. **(A)** Phenotypes of leaf tissues of *M*. ‘Profusion’ and *M. micromalus* infected by apple rust. I, II, and III represent the rust infection development stages. **(B)** Sections of disease-infected tissue. PY, pycnium; AE; aecidium; AES; aeciospores. **(C)** Morphological differences between peridial cells in different colored rust spots. **(D)** Morphological differences between aeciospore cells in different colored rust spots. Scale bars: **(A)** 1 cm; **(B)** 1.5 mm (I-II), 6 mm (III); (C) 20 μm; (D) 30 μm.

Artificially inoculated rust-infected leaves were collected (Figure 5A) and the pycnium of *M.* ‘Profusion’ were found to emerge approximately 3 days later than those of *M. micromalus*. Microscopic observations showed that the pycnium and aecidium of rust disease often co-occurred. The pycnium formed under the upper epidermis of the leaves and exposed later, and no noticeable difference was observed between the red and yellow rust spots (Figure 5B). The aecidium originated on the thick lesions on the back of the leaf and penetrated the back of the leaf; a higher number of aeciospores were observed at the yellow rust spot than at the red rust spot (Figure 5B). And tissue observation revealed that most of the peridial cells of aecidium on yellow rust spot had a complete cellular structure with tightly arranged intracellular contents. But at the red rust spots of *M.* ‘Profusion’, many of the peridial cells were distinctly irregularly crinkled, and had obvious intracellular cavities (Figure 5C). There were also marked difference in aeciospores released by the peridial cells, with distinct crinkling observed of *M.* ‘Profusion’ (Figure 5D). The morphological differences in peridial cells and aeciospores between the two *Malus* spp. may be due to differences in anthocyanin content.

### Changes in gene expression in *Malus* ‘Profusion’ and *Malus micromalus* leaves during rust infection

**Figure 6:**
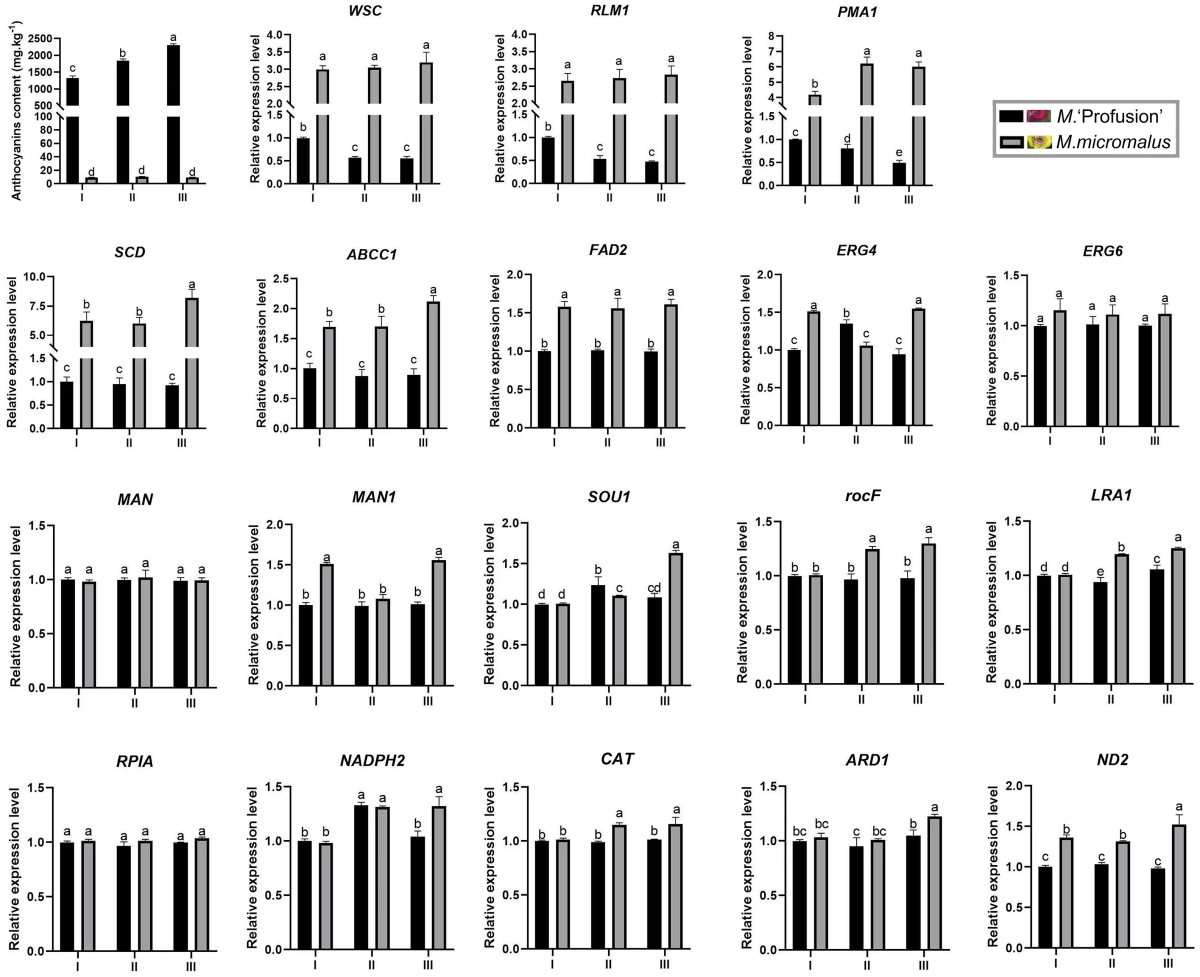
Changes in gene expression in *M*. ‘Profusion’ and *M. micromalus* leaves during rust infection. Each PCR reaction was carried out in triplicate and repeated thrice. Columns and bars represent the means and standard errors (*n* = 3), respectively.

**Figure 7:**
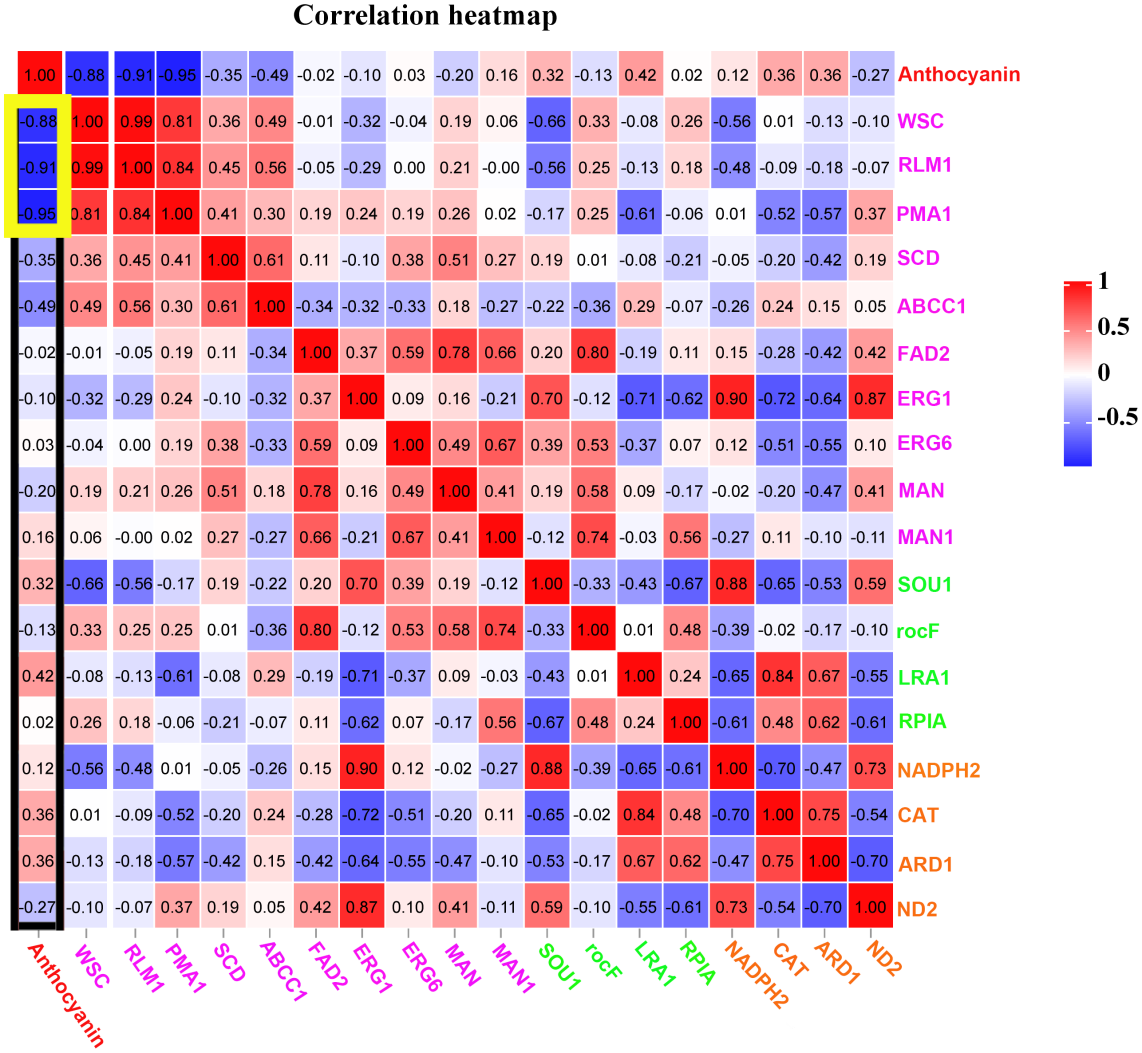
Correlations among anthocyanins and essential genes in three pathways of the cell wall and membrane metabolism, oxidoreductase activity, and antibiotic biosynthesis at rust infection development stages of RITs in *M.* ‘Profusion’. All data are representative of at least three biological replicate.

Our study revealed that the anthocyanin content of *M.* ‘Profusion’ RITs increased gradually with rust spot expansion, whereas the anthocyanin content of *M. micromalus* was extremely low and did not vary significantly (Figure 6). RT-qPCR showed that the expression levels of six key enzymes for anthocyanin biosynthesis (*PAL*, *CHS*, *CHI*, *ANS*, *DFR*, *UFGT*) differed significantly between the two *Malus* spp. The expression levels of all six enzymes were higher in *M.* ‘Profusion’ than in *M. micromalus* at different times of rust development. In *M.* ‘Profusion’, the expression levels of all five genes, except *DFR*, were progressively up-regulated as the rust spot expanded. In *M. micromalus*, the expression patterns of the six genes varied during the development of the rust. Of these, *PAL*, *CHI* and *UFGT* showed an overall up-regulation trend in expression, *CHS* and *ANS* showed similar changes, with a rising trend followed by a small decrease, and *DFR* showed a downward trend followed by a large increase (Supplementary Figure 4). Preliminary transcriptomic, physiological, and morphological studies have shown that anthocyanins inhibit *G. yamadae in vitro*, mainly by disrupting the cell wall and membrane integrity of teliospores. Tissue observations revealed significant differences in fungal cell morphology of rust spots between *M.* ‘Profusion’ and *M. micromalus*. It is unclear whether anthocyanins have a similar antifungal mechanism in *Malus* spp., DEGs for the three metabolic pathways validated in teliospores were selected for expression analysis in the RITs of *M.* ‘Profusion’ and *M. micromalus*. Most DEGs showed lower expression levels in *M.* ‘Profusion’ and higher expression levels in *M. micromalus* (Figure 6). With rust development, the expression levels of most DEGs generally increased slightly in *M. micromalus*, while *M.* ‘Profusion’ showed a different trend. Specifically, the expression of cell wall and membrane metabolic-related genes (*WSC*, *RLM1*, and *PMA1*) was gradually downregulated with the accumulation of anthocyanins. Spearman correlation analysis of anthocyanins content and gene expression in the RITs of *M.* ‘Profusion’ was carried out (Figure 7), and the results showed that the DEGs in the two pathways of antibiotic biosynthesis and oxidoreductase activity were not significantly correlated with anthocyanins. Three genes (*WSC*, *RLM1,* and *PMA1*) in the cell wall and membrane metabolic pathways were significantly negatively correlated with anthocyanins content. These data suggest that a mechanism similar to that observed *in vitro* for anthocyanins against *G. yamadae* may exist in *Malus* spp. Essential genes related to the fungal cell wall and membrane metabolic pathways (*WSC*, *RLM1*, and *PMA1*) are involved in the regulatory mechanism by which anthocyanins inhibit rust expansion in *Malus* spp.

## Discussion

Significant differences were observed in the rust susceptibility of *Malus* spp. with different colored rust spots. The incidence, RI, and lesion area ratio of *M.* ‘Profusion’ with red rust spots were significantly lower than those of *M. micromalus* with yellow rust spots. The anthocyanin content of *M.* ‘Profusion’ leaves was significantly higher than that of *M. micromalus*, especially after the onset of rust, the anthocyanins content of *M.* ‘Profusion’ increased 11.39-fold, whereas that of *M. micromalus* was extremely low, almost undetectable and also did not increase significantly after the disease. We hypothesize that the high rust resistance of *M.* ‘Profusion’ relative to *M. micromalus* may be due to anthocyanins accumulation. Previous studies have shown that anthocyanins participate in antifungal, antibacterial, and antiviral activities (Schaefer et al., 2008; Sun et al., 2018; Liu et al., 2019; Guilengue et al., 2020; Sudheeran et al., 2021). Sun et al. (2018) found that anthocyanins from wild blueberries could inhibit the growth of four foodborne pathogens, *Listeria monocytogenes*, *Staphylococcus aureus*, *Salmonella enteritidis*, and *Vibrio parahaemolyticus*. When anthocyanins were incubated with *Sclerotinia sclerotiorum* spores, spore germination and mycelial expansion were significantly inhibited, suggesting that anthocyanins affect mycelial growth (Liu et al., 2020). Anthocyanins evidence great antimicrobial potential. To evaluate the effect of anthocyanins on *G. yamadae*, we incubated different concentrations of anthocyanins from *Malus* spp. with the teliospores of *G. yamadae*. Anthocyanins effectively inhibited the germination of teliospores, and the inhibitory effect was gradually enhanced with increasing anthocyanins concentration. Inoculation experiments with teliospore suspensions treated with *Malus* spp. revealed a significant reduction in the incidence of apple rust. Based on these data, it can be concluded that anthocyanins exhibit a strong antifungal effect against *G. yamadae*.

The cell wall and membrane act as a protective barrier and maintain the integrity of the fungal cell, taking the lead in resisting stress caused by changes in the external environment. To elucidate the fungistatic mechanism of anthocyanins, we examined cell viability, as well as cell wall and membrane integrity of *G. yamadae* after anthocyanins treatment. It was found that, compared to the control, more *G. yamadae* spores were not associated with FDA fluorescence, indicating that anthocyanins severely affected the cell viability. AKP levels were significantly increased, more fungal spores were stained with PI, and electrical conductivity increased considerably after anthocyanins treatment, indicating that anthocyanins caused changes in cell wall and membrane permeability. In addition, compared with controls, anthocyanins caused marked exocytosis of the cytoplasmic material of *G. yamadae*, including proteins and nucleic acids, which is an indicator of irreversible damage to the cytoplasmic membrane and inhibition of cell survival (Shen et al., 2015). Consistent with these results, microscopic observations unambiguously confirmed severe morphological changes in fungal teliospores after anthocyanins treatment, and evident leakage of inclusions was observed. In addition to the *in vitro* tests, tissue observations showed that the fungal cells in the anthocyanin-rich *M.* ‘Profusion’ had obvious cellular crumpling compared to those of *M. micromalus*. The above evidence indicates that anthocyanins disrupt the integrity of fungal cells.

Transcriptome analysis was used to explore the molecular mechanisms of anthocyanins in *G. yamadae*. Most DEGs were associated with cell wall and membrane metabolism-related pathways. We found that *WSC* and *RLM1*, key genes in the CWI pathway, were significantly downregulated after anthocyanins treatment compared to the controls. As the main sensor of the CWI pathway, *WSC* can activate this pathway (Levin, 2005). In *Beauveria bassiana*, WSC proteins have been reported to be involved in the maintenance of fungal cell wall integrity and fungal growth (Chen, 2014). *RLM1*, a transcription factor downstream of the CWI pathway, is mainly responsible for the transcriptional activation of most genes involved in cell wall stress. In a study by Guo et al. (2020), the expression of the *RLMI* gene in *Saccharomyces cerevisiae* was downregulated under extreme stress of 0.60 g mL^−1^ sugar, and damage to the cell wall of *S. cerevisiae* was also observed, as in the present study. In addition, compared with the controls, the expression of multiple membrane-related genes was downregulated, including *PMA1*, *SLC25A23S*, *ABCC1*, *SLC35A1*, *TM9SF2* etc. Membrane proteins are responsible for various functions including nutrient transport, response to environmental stress, biofilm formation, and antibiotic resistance (Nakayama et al., 2013; Li et al., 2015). *PMA1* is a major regulator of cytoplasmic pH and plasma membrane sites in eukaryotic cells and is involved in important intracellular transport processes. PMA1 protein also can be used as a molecular target for drug discovery and provide a basis for the development of drugs for fungal diseases (Zhou et al., 2021). Transcriptome and qRT-PCR analyses showed that genes related to the cell wall and membrane changed significantly after anthocyanins treatment, suggesting that the cell wall and membrane of fungi were affected, which was consistent with our previous microscopic observations and experimental results, such as inclusion leakage. Three DEGs, *WSC*, *RLM1* and *PMA1*, were also consecutively downregulated during rust expansion in *M.* ‘Profusion’ and showed a significant negative correlation with anthocyanins content. These three genes may play key roles in disrupting the cell wall and cell membrane of *G. yamadae*.

## Conclusion

Anthocyanins effectively inhibited the germination of *G. yamadae* teliospores. After anthocyanins treatment, the cellular integrity of *G. yamadae* teliospores was destroyed, and the leakage of intercellular electrolytes, proteins, and nucleic acids increased. Results at the transcriptional level also indicated that anthocyanins effectively inhibit teliospore germination by mainly affecting the cell wall and cell membrane metabolic pathways of *G. yamadae*, in which *WSC*, *RLM1*, and *PMA1* play an essential role. In addition, obvious shrinkage was observed in peridial cells and aeciospores at the anthocyanin-rich red spots of *M.* ‘Profusion’.

## Data availability statement

The data presented in the study are deposited in the NCBI Sequence Read Archive (SRA) repository, accession number PRJNA939386: https://www.ncbi.nlm.nih.gov/bioproject/PRJNA939386.

## Author contributions

H-HL and YW planned and designed the research. YW, HA, Y-NG, QW, Y-YS, M-KC, and S-YZ performed experiments. Y-XL, J-XM, and JW analyzed data. YW and HA wrote the manuscript. All authors have read and agreed to the published version of the manuscript.

## Funding

This study was funded by the National Natural Science Foundation of China (32171862) and the Research and Development Projects in Shaanxi Province (2021NY-067).

## Conflict of interest

The author declares that the research was conducted in the absence of any commercial or financial relationships that could be construed as a potential conflict of interest.

## Publisher’s note

All claims expressed in this article are solely those of the authors and do not necessarily represent those of their affiliated organizations, or those of the publisher, the editors and the reviewers. Any product that may be evaluated in this article, or claim that may be made by its manufacturer, is not guaranteed or endorsed by the publisher.

## Supplementary material

The Supplementary material for this article can be found online at: https://www.frontiersin.org/articles/10.3389/fmicb.2023.1152050/full#supplementary-material

## Abbreviations

ABCC1: ATP-binding cassette, subfamily C (CFTR/MRP), member 1
ACAA1: acetyl-CoA acyltransferase 1
ALG11: alpha-1,2-mannosyltransferase
ANS: anthocyanin synthase
ARD1: D-arabinitol dehydrogenase
ARF1: ADP-ribosylation factor 1
CAT: catalase
CHI: chalcone isomerase
CHS: chalcone synthase
DFR: Dihydroflavonol 4-reductase
ERG4: Delta24(24(1))-sterol reductase
ERG6: sterol 24-C-methyltransferase
ERG25: methylsterol monooxygenase
fabG: 3-oxoacyl-[acyl-carrier protein] reductase
FAD2: omega-6 fatty acid desaturase / acyl-lipid omega-6 desaturase (Delta-12 desaturase)
GMPP: mannose-1-phosphate guanylyltransferase
LRA1: L-rhamnose 1-dehydrogenase
MAN: mannan endo-1,4-beta-mannosidase
MAN1: mannosyl-oligosaccharide alpha-1,2-mannosidase
MNN2: alpha 1,2-mannosyltransferase
NADPH2: NADPH2 dehydrogenase
ND2: NADH–ubiquinone oxidoreductase chain 2
OPR: 12-oxophytodienoic acid reductase
PAL: phenylalanine ammonia lyase
PMA1: H^+^-transporting ATPase
POR: NADPH-ferrihemoprotein reductase
RLM1: MADS-box transcription factor
rocF: arginase
RPIA: ribose 5-phosphate isomerase A
SCD: stearoyl-CoA desaturase (Delta-9 desaturase)
SLC25A23S: solute carrier family 25 (mitochondrial phosphate transporter), member 23
SLC35A1: solute carrier family 35 (UDP-sugar transporter), member A1
SOU1: sorbose reductase
TM9SF2: transmembrane 9 superfamily member 2
UBP1: ubiquitin carboxyl-terminal hydrolase 1
UFGT: UDP-glucose: flavonoid 3-O-glucosyltransferase
WSC: cell wall integrity and stress response component
