# Supplementary material for: Anthocyanins from Malus spp. inhibit the activity of Gymnosporangium yamadae by downregulating the expression of WSC, RLM1, and PMA1
Source: Front Microbiol. 2023 May 3;14:1152050. doi: 10.3389/fmicb.2023.1152050 (PMC10191115; doi:10.3389/fmicb.2023.1152050)
Supplement: Supplementary file 1 [file Data_Sheet_1.pdf]

Supplementary Table 1 Quantitative real-time PCR primer sequence.

Gene name	Direction	Sequence(5'-3')
ARF1	Forward primer	TTAGCCCCAAAATCCCAT
	Reverse primer	ACACCCGTGAGAAGAACC
18S	Forward primer	GTCACTACCTCCCCGTGTCA
	Reverse primer	GAGCCTGAGAAACGGCTACC
WSC	Forward primer	ATGGTGCCTCGCCGCTTTGGCCTGT
	Reverse primer	ACAGGCCAAAGCGGCGAGGCACCAT
RLM1	Forward primer	ATGTGGCTGTCATGTCGTCCTTCCA
	Reverse primer	CCTGGCACTGCGTCAAAGAAC
PMA1	Forward primer	ATGGCAGGAGCGATCATGAAGAACG
	Reverse primer	CGGTTTCTCCTCTGCCTCA
SCD	Forward primer	ATGATTTTTCTCTTGCACAAATACA
	Reverse primer	CCAGCGGCGTGTATTTGTGC
ABCC1	Forward primer	ATCAGAAACTGTCTGAACGGCAAA
	Reverse primer	CCAGCAGGAATGTGGAAGC
FAD2	Forward primer	CCTCTTCCCCAGACTCCATTGATGC
	Reverse primer	CCTGGGGCAGTGCTGTTGAGGTCAT
ERG4	Forward primer	ATGTCTACCGTCATCTGTGACCGGC
	Reverse primer	GACCACCGAATGAAGCACGAA
ERG6	Forward primer	CGAAACAGGACGAGAATAAGCG
	Reverse primer	TAGTAGTTTAGTGATTGCGGGCAT
MAN	Forward primer	AAAAGCGGAAGCAAGCTCGCATTTC
	Reverse primer	CTTGTGAGGGAGACGAGCATC
MAN1	Forward primer	ATGACTGGTAATGCTGATGCGAGAT
	Reverse primer	ATCTCGCATCAGCATTACCAGTCAT
SOU1	Forward primer	ATGCGTACCCCCACAAACGAGCTTC
	Reverse primer	CGGTGAAGGCGGGTGGAA
rocF	Forward primer	TTTGAAGCTTTCTCCCATTGGGAAA
	Reverse primer	TGGATGGGCTGGAAGACAAAA
LRA1	Forward primer	GGGAATCCGAGCCTGGTAG
	Reverse primer	AGGCTGTTTGGACTGGGGGTGACAT
RPIA	Forward primer	ATGGCCCCGCTCTACAACACCTTCT
	Reverse primer	GGGCTTTGAGTGGAAGGGAG
NADPH2	Forward primer	ATGGAGCCCTGTCTGGCAAAGGCAG
	Reverse primer	TGGCTTCCCTGGCGGTGT
CAT	Forward primer	ATGCCAGCCAAAAACGTCCATGAAA
	Reverse primer	TTTCATGGACGTTTTTGGCTGGACT
ARD1	Forward primer	GCTATCCTTGCCAATCCAGAGCCCA
	Reverse primer	TGGTGGGCTCTGGATTGG
ND2	Forward primer	GTTGGGAGGAAAGCGGTAGGGTAAG
	Reverse primer	CTTACCCTACCGCTTTCCTCCCAAC

Supplementary Table 2 Summary of the sequencing data from control and anthocyanin-treatment groups.

Sample_ID	Total Raw Reads (M)	Clean_Reads(M)	Clean_Bases(Gb)	Clean Reads Q20(%)	Clean Reads Q30(%)	Clean Reads Ratio(%)
Control_1	43.82	42.04	6.31	96.23	90.81	95.93
Control_2	45.57	43.29	6.49	96.47	91.35	94.98
Control_3	45.57	43.55	6.53	96.27	90.87	95.55
Anthocyanin treatment_1	45.57	43.38	6.51	96.59	91.56	95.18
Anthocyanin treatment_2	43.82	42.23	6.34	96.65	91.7	96.38
Anthocyanin treatment_3	45.57	43.59	6.54	96.64	91.69	95.64

Supplementary Table 3 The significant enrichment terms in the groups of control VS anthocyanin-treatment.

Term type	GO Term ID	GO Term	Term candidate gene number	Term gene number	P value
molecular_function	GO:0004377	GDP-Man:Man3GlcNAc2-PP-Dol alpha-1,2-mannosyltransferase activity	4	9	2.05E-06
	GO:0016491	oxidoreductase activity	20	638	5.18E-05
	GO:0050577	GDP-L-fucose synthase activity	2	6	1.92E-03
	GO:0004129	cytochrome-c oxidase activity	3	28	3.98E-03
	GO:0016594	glycine binding	2	2	1.32E-04
	GO:0003746	translation elongation factor activity	7	113	3.34E-04
	GO:0016985	mannan endo-1,4-beta-mannosidase activity	4	35	6.75E-04
	GO:0016767	geranylgeranyl-diphosphate geranylgeranyltransferase activity	2	5	1.29E-03
cellular_component	GO:0016021	integral component of membrane	114	6825	9.00E-05
	GO:0005615	extracellular space	3	32	7.52E-03
	GO:0005960	glycine cleavage complex	2	8	4.23E-03
biological_process	GO:0006696	ergosterol biosynthetic process	4	76	1.08E-02
	GO:0016126	sterol biosynthetic process	3	43	1.26E-02
	GO:0030488	tRNA methylation	3	43	1.26E-02
	GO:0000086	G2/M transition of mitotic cell cycle	2	15	1.21E-02
	GO:0016117	carotenoid biosynthetic process	3	6	2.78E-05
	GO:0006080	substituted mannan metabolic process	4	20	6.67E-05
	GO:0002128	tRNA nucleoside ribose methylation	2	6	1.85E-03
	GO:0009226	nucleotide-sugar biosynthetic process	2	6	1.85E-03

	GO:0046020	negative regulation of transcription from RNA polymerase II promoter by pheromones	2	9	4.35E-03

Supplementary Table 4 The significant enrichment terms in the groups of control VS anthocyanin-treatment.

Term type	KEGG Pathway ID	KEGG Pathway name	Term candidate cene number	Term gene number	P value
Environmental Information Processing	ko04011	MAPK signaling pathway	38	1425	1.05E-04
Genetic Information Processing	ko03450	Non-homologous end-joining	6	212	8.23E-02
	ko03410	Base excision repair	7	175	1.26E-02
	ko03040	Spliceosome	18	790	3.28E-02
Cellular Processes	ko04146	Peroxisome	8	320	8.69E-02
	ko04139	Mitophagy	7	252	6.87E-02
Metabolism	ko00510	N-Glycan biosynthesis	6	211	8.09E-02
	ko01212	Fatty acid metabolism	6	208	7.67E-02
	ko00040	Pentose and glucuronate interconversions	5	163	8.32E-02
	ko00100	Steroid biosynthesis	3	74	8.83E-02
	ko00906	Carotenoid biosynthesis	3	6	5.49E-05
	ko00051	Fructose and mannose metabolism	8	188	5.49E-03
	ko00513	Various types of N-glycan biosynthesis	7	175	1.26E-02
	ko00592	alpha-Linolenic acid metabolism	3	44	2.45E-02
	ko00254	Aflatoxin biosynthesis	2	17	2.38E-02
	ko00410	beta-Alanine metabolism	5	129	3.70E-02

	ko01040	Biosynthesis of unsaturated fatty acids	4	90	3.93E-02
	ko01130	Biosynthesis of antibiotics	24	1191	5.20E-02
	ko00380	Tryptophan metabolism	5	141	5.09E-02
	ko00010	Glycolysis / Gluconeogenesis	7	269	8.97E-02

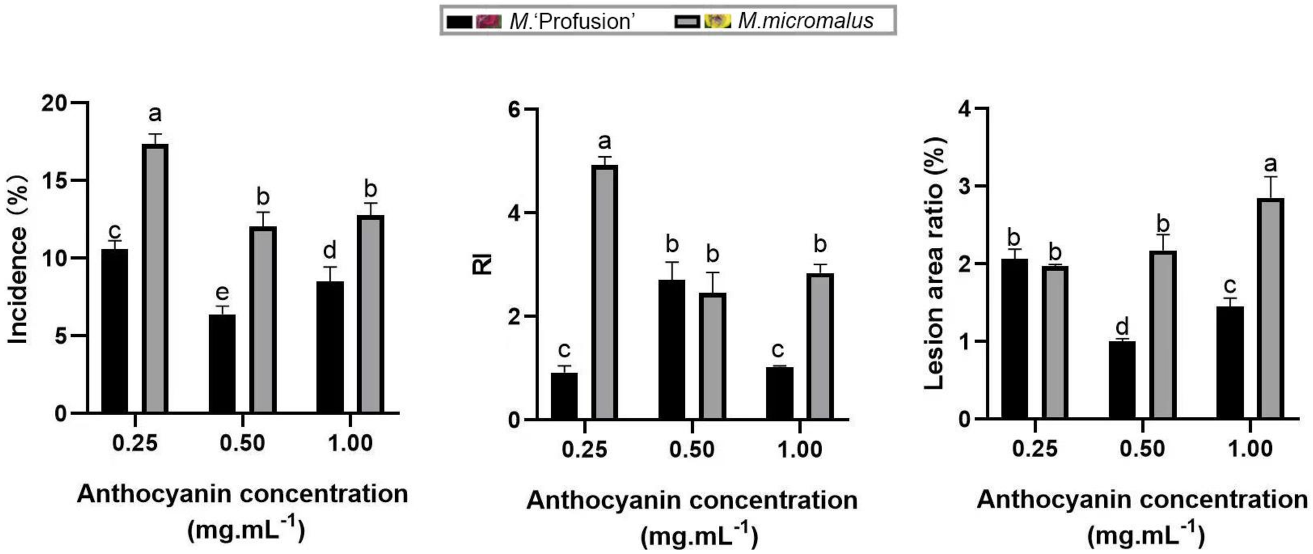

Supplementary Figure 1. Rust infection severity of *Malus* spp. inoculated with anthocyanin-treated teliospores suspensions.

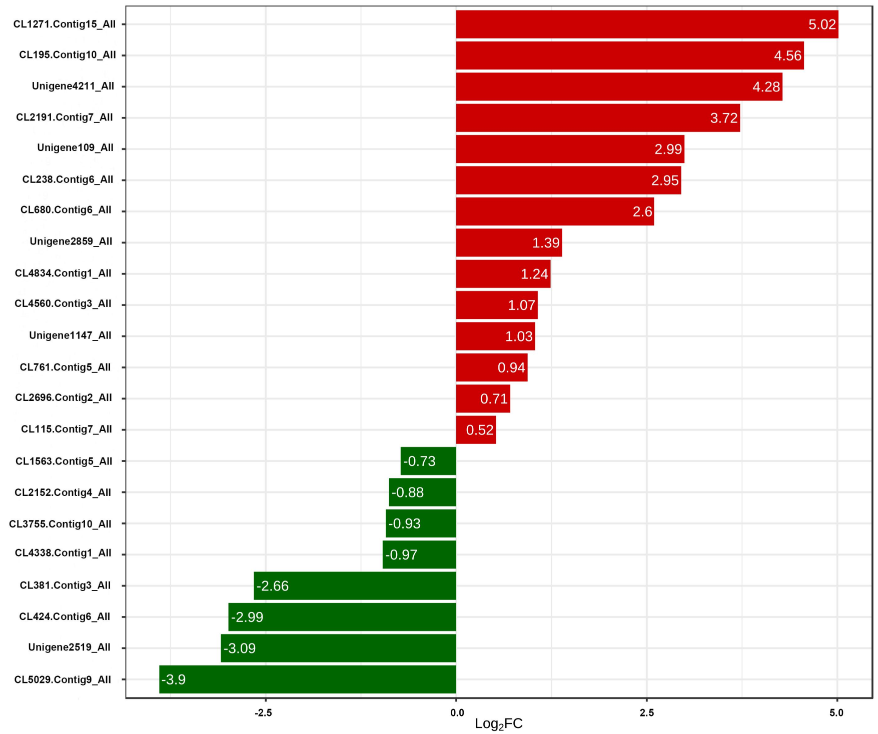

Supplementary Figure 2. Comparative analysis of cell wall and cell membrane-related DEGs in *G. yamadae*. Red indicates upregulated genes, and green indicates downregulated genes.

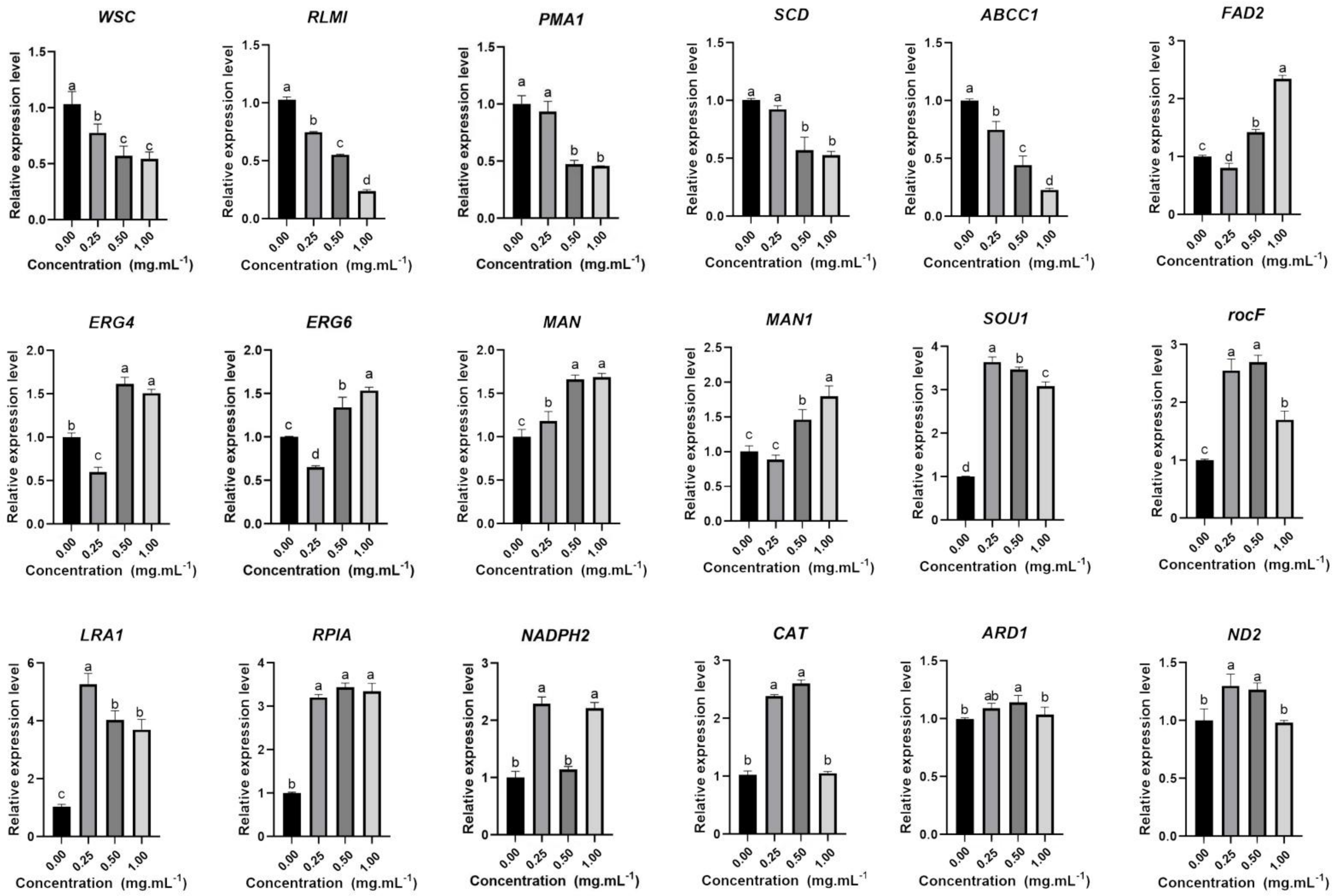

Supplementary Figure 3. Relative expression levels of candidate DEGs according to the quantitative real-time PCR. Each PCR reaction was carried out in triplicate and repeated thrice. Columns and bars represent the means and standard errors (n = 3), respectively.

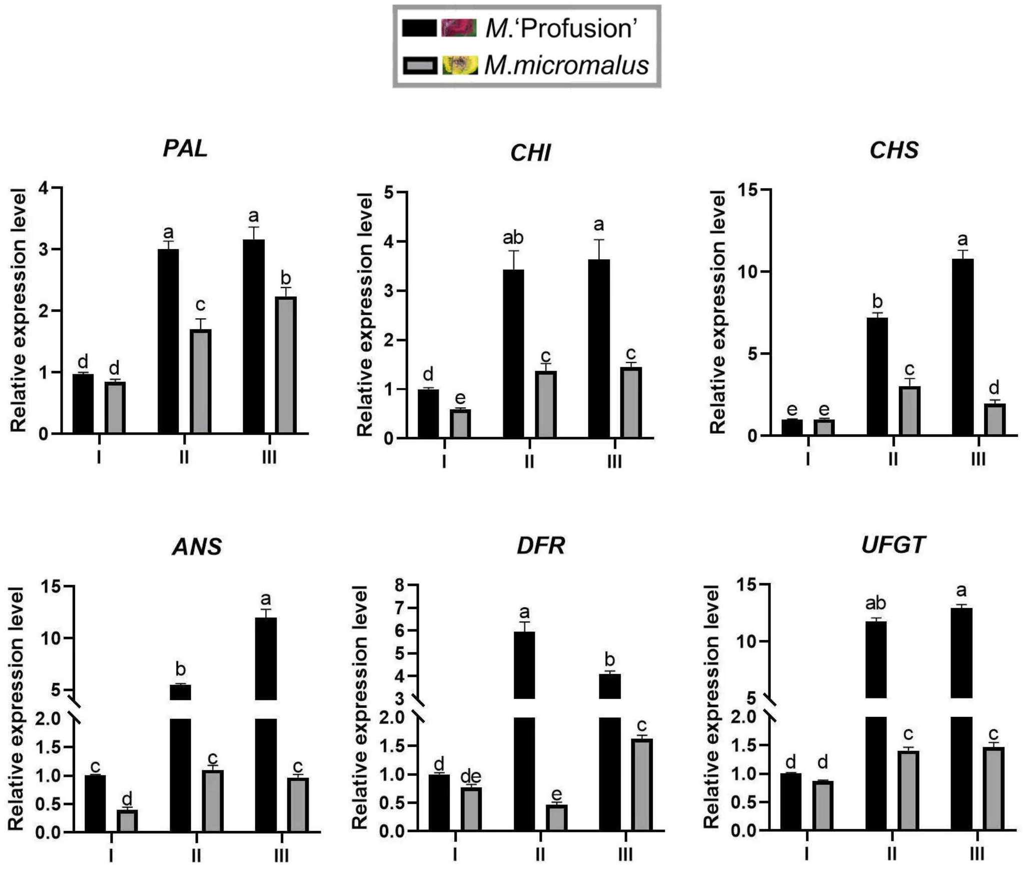

Supplementary Figure 4. Relative expression profiles of anthocyanin biosynthesis genes in *M.* 'Profusion' and *M. micromalus* leaves during rust infection. Each PCR

reaction was carried out in triplicate and repeated thrice. Columns and bars represent the means and standard errors (n = 3), respectively.
